# Understanding the Unmet Needs of People Living with Type 2 Diabetes in Self-Managing Their Condition

**DOI:** 10.3390/nu17071243

**Published:** 2025-04-02

**Authors:** Katerina Sarapis, Yingting Cao, Melissa Abou Chakra, Jack Nunn, Pradeep Rathod, Mark Weber, Carlyle Albuquerque, Maryse Chapman, Richard Barr, Christopher Gilfillan, Helen Skouteris, Brian Oldenburg, Peter Brukner, Alison Beauchamp, George Moschonis

**Affiliations:** 1 Discipline of Food, Nutrition and Dietetics, Department of Sport, Exercise and Nutrition Sciences, School of Allied Health, Human Services and Sport, La Trobe University, Bundoora, VIC 3086, Australia; k.sarapis@latrobe.edu.au (K.S.); tina.cao@latrobe.edu.au (Y.C.); jack.nunn@latrobe.edu.au (J.N.); pradeep.rathod@hotmail.com (P.R.); weebs@ozemail.com.au (M.W.); carlyle8899a@hotmail.com (C.A.); marysecee@gmail.com (M.C.); richardjbarr@yahoo.com.au (R.B.); 2 Implementation Science Lab, Baker Heart and Diabetes Institute, Melbourne, VIC 3004, Australia; brian.oldenburg2@baker.edu.au; 3 School of Rural Health, Monash University, Clayton, VIC 3800, Australia; melissa.abouchakra@monash.edu; 4 Endocrine Research, Eastern Clinical Research Unit, Eastern Health, Melbourne, VIC 3128, Australia; chris.gilfillan@easternhealth.org.au; 5 Monash Centre for Health Research and Implementation, School of Public Health and Preventive Medicine, Monash University, Melbourne, VIC 3168, Australia; helen.skouteris@monash.edu; 6 School of Psychology and Public Health, La Trobe University, Bundoora, VIC 3086, Australia; 7 La Trobe Sport and Exercise Medicine Research Centre (LASEM), School of Allied Health, Human Services & Sport, La Trobe University, Bundoora, VIC 3086, Australia; p.brukner@latrobe.edu.au; 8 La Trobe Institute for Sustainable Agriculture & Food (LISAF), La Trobe University, Bundoora, VIC 3086, Australia

**Keywords:** consumer involvement, type 2 diabetes, unmet needs, lived experience, self-management, solutions

## Abstract

**Background/Objectives:** Type 2 diabetes (T2D) prevalence is rising worldwide. Despite numerous efforts to address the condition, many initiatives fall short due to limited consumer engagement. Involving people with lived experience in healthcare design is increasingly recognized as an effective strategy for improving diabetes management. **Aim:** To understand the unmet needs of people with T2D in self-managing their condition through the establishment of a Consumer Reference Group (CRG). **Methods:** The CRG was established using a standardized approach advised by Monash Partners, with specific terms of reference for consumer engagement. A face-to-face training workshop was conducted to develop consumers’ capacity to co-design T2D interventions. Two focus groups were held to explore consumers’ unmet needs and propose potential solutions. An inductive thematic analysis was performed. **Results:** Ten adults (three females/seven males; 58–78 years old) with T2D participated. Four main themes emerged: (1) misinformation; (2) limited guidance; (3) challenges in self-management; and (4) gaps in prevention and screening. Participants reported difficulties in maintaining motivation, balancing T2D management with other life commitments, and addressing mental health concerns. They reported feeling misinformed and inadequately supported by healthcare professionals and diabetes organizations, often relying on conflicting sources of information. Participants from culturally and linguistically diverse (CALD) backgrounds noted a lack of tailored dietary information. Proposed solutions included better training for primary care providers, reinforcing the role of diabetes nurse educators, expanding mental health support, and collaborating with CALD communities to provide culturally appropriate dietary information. **Conclusions:** These insights are critical for developing consumer-driven interventions that are responsive to the real-world needs of people living with T2D.

## 1. Introduction

Diabetes is a long-term chronic disease characterized by high blood glucose levels and is associated with serious complications and co-morbidities [1]. In 2021, diabetes was the eighth leading cause of combined death and disability worldwide, with almost 537 million people (aged 20–79 years) living with the disease, while type 2 diabetes (T2D) accounted for 90% of all diabetes cases [2]. In Australia, over 1.3 million adults—about 1 in 20—were living with T2D in 2022 [3], although between 2000 and 2021, the age-standardized incidence rate decreased by 43% [4]. This might be due to improved screening and early interventions [5], increased awareness of diabetes risk factors [6], as well as public health initiatives targeting obesity and metabolic health [7]. However, it should be noted that disparities still exist among certain populations, including lower socioeconomic groups [4]. Several other countries have also reported a decline in T2D incidence [8,9], which may be attributed to improved preventive strategies that promote behavioral changes and risk factor modification [8]. However, despite the decline in T2D incidence observed in Australia and some other countries, the burden of the disease remains particularly high, while predictions are also discouraging, considering that 783 million people are expected to be diagnosed with T2D in 2045, indicating an increase of 46% since 2021 [10].

Managing T2D is recognized as challenging for both patients, their carers, and healthcare providers. The 2019 American Diabetes Association (ADA) and the European Association for the Study of Diabetes (EASD) consensus report, which proposed strategies for managing T2D, highlighted the importance of comprehensive disease risk management and patient-centered care, with the aim of reducing complications and maintaining quality of life (QoL) [11]. Patient-centered care focuses on recognizing individual uniqueness and emphasizes the importance of healthcare providers working with individuals (also referred to as “consumers”) to make shared decisions [12]. Failing to include consumers’ shared needs, values, and preferences through person-centered care is likely to reduce the effectiveness of healthcare, both in the short and long term [13].

Research has identified that inadequate support from healthcare systems in diabetes education and self-management [14,15,16,17,18] plays a crucial role in self-care behaviors, such as influencing people’s motivation for self-management of their health condition [19,20]. Other studies have also demonstrated that lack of knowledge about disease management, difficulties in accessing community resources, and poor health literacy are key barriers to the implementation of self-management strategies for individuals living with T2D, including culturally and linguistically diverse (CALD) populations [21,22]. Influencing and sustaining long-term behavioral changes is challenging; therefore, understanding the unique perspectives on the enablers and barriers to the uptake of self-management strategies can allow health professionals to deliver tailored interventions rather than relying on one-size-fits-all models of care [23,24,25]. 

Both evidence and experience demonstrate that direct involvement of consumers and communities in healthcare, i.e., through the establishment of consumer and community involvement committees or advisory groups (reference group), has the potential to integrate consumer and healthcare provider experiences’ (as well as those of other stakeholders) in order to identify effective strategies that will align with consumer needs [26]. Engaging communities effectively can assist health services by providing insights/advice on consumer unmet needs and active participation in health service development, planning, and quality improvement [27]. Crawford et al. [28] suggest that involving consumers can make health services more accessible and acceptable, ultimately improving health outcomes and QoL. Additionally, Boote et al. [29] highlight that consumer involvement can also enhance the quality and relevance of health research since they offer their personal experience and view the research/project from a different lens than health professionals and researchers. While Whitstock [30] emphasizes that it can also lead to better adoption of research findings in clinical practice. This holistic approach underscores the importance of collaboration between healthcare providers, researchers, and patients/consumers to ensure that services and research are aligned with their needs and preferences [13].

Improving self-management of T2D is crucial for reducing morbidity, mortality, and healthcare costs, but gaps still remain in the system’s ability to support this. Therefore, consumer-led decision-making, supported by tools that illustrate the benefits and risks of different treatment options, alongside careful consideration of cultural, psychosocial, and other characteristics, as well as people’s preferences, is essential in tailoring T2D treatment goals and strategies. The objectives of the current study were to (1) understand consumers’ unmet needs in self-managing their condition through the development of a Consumer Reference Group (CRG) comprising people living with T2D in Australia, and (2) actively engage consumers in the identification of solutions for T2D management that will eventually drive the co-design of effective interventions tailored to their unique needs.

## 2. Materials and Methods

The development of the CRG followed a standardized approach, as advised by Monash Partners for developing a CRG from scratch [31]. This involved a transparent and open recruitment process with the application of a set of selection criteria for the identification of eligible consumers and specific terms and conditions of their engagement. To facilitate methodological transparency and repeatability, the process for the development of the CRG was registered in the Standardised Data on Initiatives (STARDIT) platform (https://stardit.wikimedia.org.au) (accessed on 19 November 2024). The study was conducted in accordance with the Declaration of Helsinki, and the protocol was approved by the Human Research Ethics Committee of La Trobe University (HEC23452). Informed consent was obtained from all participants involved in the study.

### 2.1. Sampling Procedure for Participation in the Consumer Reference Group (CRG)

A purposive sampling approach was used to identify people with lived experience of T2D who met the eligibility criteria to be included in the CRG. The establishment of the CRG initially involved the setup of an Interim Reference Group (IRG), where researchers from La Trobe University met with key stakeholders and consumer representatives from Diabetes Australia, the Defeat Diabetes Program, and Eastern Health. The scope of the meeting was to define the preferred characteristics of consumers, the recruitment criteria, and the terms of reference (TOR), which clearly state the purpose and structure of the committee, its objectives and scope, roles, and responsibilities. Expressions of interest were then advertised (i.e., via newsletters, flyers, email, and word of mouth) through the networks of people with T2D in Diabetes Australia, Defeat Diabetes Program, and Eastern Health. An online questionnaire was used during the screening phase to identify potentially eligible participants (CRG members). Inclusion criteria were (1) adults aged ≥ 18 years with lived experience with T2D; (2) all gender identities, including men, women, LGBTIQA+, and others; (3) the ability to communicate (read and understand) in English; (4) access to the internet and smartphone; and (5) residency in Victoria (to be able to join in-person sessions). Prior to participation, all eligible participants provided their signed consent.

### 2.2. Training Workshop

Following the establishment of the CRG, a face-to-face training workshop (4 h) was conducted at La Trobe University to help consumers build skills that will empower them to engage effectively in the development of interventions tailored to their needs. More specifically, the training aimed to build consumers’ capacity and independence in research by moving away from a tokenistic representation of consumers to an approach that would allow them to be actively engaged across all stages of the research process (i.e., framing the research questions, the co-design, implementation, and evaluation of the effectiveness of the intervention). The design of the training workshop was based on a consumer training course developed by the Telethon Kids Institute [32]. The skills consumers will gain through training will not only enable the establishment of an equal partnership between consumers and researchers in all stages of the research process, but also empower their active involvement in policymaking, health service development, planning, and quality improvement initiatives. The ultimate goal is the development of effective self-management interventions in response to the identified unmet needs of people with T2D [33,34]. A detailed description and the content of the training workshop can be found in the Supplementary Material File S1.

### 2.3. Focus Groups

Two focus groups were conducted with the same participants after the training workshop, to gain an in-depth understanding of consumers’ unmet needs in self-managing their health conditions and to identify solutions that might facilitate the design and implementation of effective intervention programs, with their leading engagement in all stages of their design, implementation, and evaluation. Both focus groups were conducted to cover at least 80% of data saturation [35], and the reporting of this study follows the “Consolidated criteria for reporting qualitative research (COREQ): a 32-item checklist for interviews and focus groups” [32].

The first focus group was performed face-to-face and had a total time duration of two hours, while the second one was held via Zoom and lasted one hour. Broad topics for the focus group discussion were determined a priori by the researchers based on the aim/purpose of establishing the reference group, supported by previous evidence [13]. Each focus group was facilitated by an experienced qualitative researcher (AB) who had no prior relationship with participants. The facilitator first asked participants to describe what self-management of their T2D meant to them in terms of the required activities, before asking what skills and resources people need to undertake these activities. Participants were then asked to describe unmet needs in relation to self-management and suggest any recommendations for how to address these unmet needs. Findings were summarized and presented to the same group of participants in a second focus group one month later; firstly, as a way of data validation through member checking, and secondly, to prompt further discussion about unmet needs and potential solutions to those unmet needs. Participants were offered AUD 400 in gift cards as a token of appreciation for their participation in the focus groups.

### 2.4. Data Analysis 

Focus groups were audio-recorded and transcribed verbatim. An inductive thematic analysis approach was used to analyze the data and identify themes, based on Braun and Clarke’s 6-step framework [36]. After familiarizing themselves with the data, two researchers (AB, MAC) independently coded the data using NVivo^®^ software (version 14). The process involved initial line-by-line coding using an open coding approach to describe the data. Codes were continuously developed and modified throughout the coding process by the researchers. The two researchers then compared codes and modified them by consensus, before independently generating themes and sub-themes that were considered to represent the unmet needs of participants. Two cross-checks to compare emerging themes were performed to assess the accuracy of inferences. Where a difference was found, the researcher was asked to demonstrate from the raw data how their interpretation was reached until agreement was reached.

## 3. Results

From 25 individuals who completed the online screening questionnaire, ten adults aged 58–78 years (three females, seven males) living with T2D (mean duration 17.7 years) were included in the CRG, attended the training workshop, and participated in the focus groups. The final selection was based on ensuring diversity in lived experiences, willingness to engage in a structured CRG, and an expressed interest in ongoing involvement and co-designing diabetes management programs. Out of the ten consumers, four were Australian, three were Asian/Pacific Islanders, two were SE European and SW European, and one was North American; six had tertiary education (University) and four had post-secondary qualifications (TAFE, diploma). All consumers were living in Victoria. 

From the thematic analysis, a total of four themes and 13 sub-themes were identified. The list of themes and sub-themes is presented in Table 1.

### 3.1. Theme 1: Need for Guidance

#### 3.1.1. Role of Health Professionals

Focus group participants stressed the importance of having knowledgeable and proactive health professionals involved in their care:

*“If you’re on meds, you have to have a really good professional who understands your metabolism and everything that’s going on within you, order the right tests and carry out a gamut of tests.”*

However, some participants reported difficulty in finding a healthcare professional who is up to date with the information they provide and has time to spend with them:

*“Finding GPs [General Practitioners] who have time and knowledge to tackle this issue is a major problem.”*

Participants also noted that multiple health professionals were involved in their care:

*“…because diabetes affects so many different parts of the body. So, endocrinologists, ophthalmologists, cardiologists.”*

It was generally agreed that patients benefited the most from the guidance of diabetes educators and diabetes nurses: *“I found the nurse more relevant to her knowledge”*. Participants suggested that these health professionals could be more actively involved in care by taking on a role as the primary provider and care coordinator for the management of their T2D, which may counteract some of the challenges mentioned above:

*“Your diabetes education nurse should be your primary focus, and she should be the person that you, he or she, should be the person that you visit regularly. And direct, you work with them to see when you, which specialist you need to see, which GP you need to see…it has to be the diabetes educator, nurse model, that should be your primary care provider.”*

Role of Diabetes Associations

In Australia, there are a number of national and state associations (i.e., not-for-profit) that offer a range of resources and advocacy support to people with T2D, their carers, and clinicians [37,38]. In terms of lived experiences with these associations, participants had mixed feedback. While some participants considered the associations as credible sources of information, others felt that the information provided was not relevant to their condition:

*“We’re carbohydrate intolerant, but they are recommending 5 serves of carbs a day.”*

Several participants considered that a major role for diabetes associations was to provide more support to people with T2D who were from culturally and linguistically diverse (CALD) backgrounds:

*“But we really need to consider our culturally and linguistically diverse populations”.*

*“True.”*

*“Huge issue. [Associate name] wasn’t, hasn’t been helpful, that’s an interesting point.”*

However, others noted that translated resources are available:

*“I think [Association name] have got translation in various languages as well.”*

It was also thought that these associations could take a stronger advocacy role in the prevention of diabetes through raising awareness of diabetes in the community, for example, through public health campaigns:

*“You have a Shave for Cancer Day for example, if [Association name] said, why don’t we have a Give up Sugar for one day, just promote those sorts of things, get people thinking about it.”*

This advocacy role also included reaching out to cultural leaders who can influence CALD communities to find resources and uptake information:

*“And if they tap into cultural leaders, who could influence peoples and uptake of the information, and, also then if these people are present, they can talk to individuals.”*

#### 3.1.2. Diet Management

Participants reported an overall lack of guidance in managing a diabetic diet. When asked about managing their diet after being first diagnosed with T2D, participants expressed that they only had limited information, which led to a fear of restrictions:

*“* *When you’re first diagnosed you sort of, you have a basic awareness of what’s required. Then there’s that sort of, oh my god I’m never going to be able to have ice-cream again, which sounds pathetic but it’s just, that sort of sense of what would you call it, impending doom.”*

Because of this lack of guidance, people had to try different diets before finding a diet that worked for them:

*“Low carb, high protein, fasting, every different diet that you can think of.”*

However, participants agreed that while it does take time to learn, it is not difficult to maintain:

*“Once you get the hang of a low carb diet it’s fairly easy to maintain it.”*

Some participants were not sure what a low-carbohydrate diet is or what 30 g of carbohydrates looks like. Other participants reported having to find their own information online while trying to understand the glycaemic index and glycaemic load.

The cultural aspects of managing diet were also highlighted, with participants noting that dietary guidelines must acknowledge the diversity of foods and diets within Australia:

*“But you need those guidelines to be culturally rich because you’ve got bread, roti, you’ve got noodles, you’ve got some of the Greek guys here, maybe something that’s food.”*

However, participants noted that there was a general lack of culturally tailored guides and resources for people from a culturally and linguistically diverse (CALD) background:

*“Information about this is non-existent, right. Vegetarian, no carb diet.”*

### 3.2. Theme 2: Ambiguous and Conflicting Information

#### 3.2.1. Information from Health Professionals and Other Sources

Participants reported receiving incorrect information about self-management from healthcare providers. For instance, they were told by a health professional that they only need to check their blood sugar once a day or received conflicting information from different clinicians:

*“There’s a lot of conflicting information…Conflicting information between professionals, medical professionals sometimes.”*

Participants also raised the issue that while specialists tend to be current with the latest evidence, this is not always the case for all primary care providers. One participant suggested the need for a *“re-education of anybody in diabetes field”*. This mistrust around the currency and accuracy of information from health professionals meant that some participants resorted to the internet to build their knowledge. However, participants expressed difficulty in finding credible sources of information online and in trusting the accuracy of that information:

*“Most people, most reporters have biases, that some articles can be tainted with personal views, just as YouTube, it’s a source of information, it’s like a newspaper. I’d be very skeptical, I’d check and double-check…”*

#### 3.2.2. Diet Misconceptions

As mentioned earlier, keeping up with a healthy diet after a diabetes diagnosis can be daunting, especially without the right help and navigation. Participants showed an understanding of the basics of a low-carbohydrate diet:

*“Smaller, smaller portion [of carbs].”*

However, having to find this information independently led to misinformation and some incorrect beliefs. One of the biggest misconceptions was thinking that they would not be able to enjoy an occasional sweet treat or a fast-food meal every once in a while: *“No junk food basically”*, or that many foods would have to be completely cut down from their diet:

*“I don’t think I can live the rest of my life with no potato, rice, bread.”*

Another example of a misconception was seen around carbohydrates:

*“Myth or is it a reality that if these starchy foods are cooked and then refrigerated then the type of starch changes. And that starch is then unabsorbable in someone?”*

It should be acknowledged that participants’ perspectives may have been shaped by prior engagement in structured diabetes programs, including those advocating for lower-carbohydrate dietary approaches.

#### 3.2.3. The Food Industry

Participants reported a perception that the food industry was a major contributor to misinformation about diet.

*“The food industry is a powerful lobby group”*. They discussed that some industries may only advertise ‘the healthy side’ of their products or spread misinformation about other industries and companies to promote their sales:

*“So say that this group has been funded by the Cattle Industry Association. And we are all eating beef and a nice low carb diet to control diabetes, but on the other side there’s a [company name] group that’s been funded by [company name] and they are coming out and saying, if you eat beef, you will get heart disease. If you eat beef, you will get dementia. If you eat beef, you will have heart pressure, you will get stroke.”*

There was also a general consensus among participants that food products contain too much sugar and that limits should be imposed:

*“I don’t think they are regulated on that. So all products, all food, all drinks, they contain sugar so much and nobody tells them, guys you’re far above the limit.”*

Food labeling was also noted as an issue: *“Labelling is nightmare”*. Challenges included the composition of some food products, where the general population cannot recognize or read most ingredients: *“Ingredients we don’t recognize it’s all artificial”*. Those ingredients are usually written in a very small font, making you need a *“set of glasses every time you try to read it”*.

Participants agreed that food packaging should be more transparent when it comes to the composition of the product, mainly sugars, and that regulations should be more rigid. A suggested solution was a national approach to food labels consisting of a traffic light system. This means the product would display a green sign when suitable for people with diabetes, orange or yellow when it should be consumed in moderation, and red when individuals with diabetes should avoid it completely:

*“So they might have red for no go, orange for maybe, and green for go.”*

### 3.3. Theme Three: Mental Health

#### 3.3.1. Managing Negative Emotions

Participants reported a sense of hopelessness regarding their ability to cope with the requirements of diabetes self-management, and a fear of failing and then having to deal with the consequences:

*“Yes, the hopelessness comes from a number of points. A) is the realization that it is going to be a pretty long-term disease or long-term condition. Generally speaking, it keeps getting worse. So your medication, the need for medication keeps increasing, even though you might be looking after yourself well…So there’s a downward progression of the disease. And so the hopelessness is that I am going to get amputation, or I am likely to get heart disease, or I am likely to get Alzheimer’s or dementia. And there is nothing I can do.”*

Participants reported limited confidence that they are completely on top of self-management:

*“Reasonable but not great”.*

Participants also reported feeling guilty about not adhering to recommendations around self-management, but recognized that they were mostly trying the best they could, and that guilt was not helpful:

*“But we’ve got to not beat ourselves up and try and do it more times. I think, we’ve very quick, like you just commented to me, don’t get down on yourself. I do get lazy with it, and I think that’s what it is. I’m going okay, I’m healthy, but I have diabetes.”*

#### 3.3.2. Role of Others

Participants reported an unmet need for mental health support from trained professionals who also understood the complexities of living with a chronic condition, such as diabetes:

*“I reckon mental health support is a big one…to get a professional who is also good at understanding diabetes and what are the mental health issues of managing a chronic condition over a very long period of time that’s, I think is that unmet need at the moment for quite a lot of people.”*

Support from family and friends was limited, often because people do not understand what living with diabetes entails:

*“So, I often tell people that, I have got diabetes, and they just shake their head and say, okay. But they really have no understanding what it is.”*

#### 3.3.3. Learning to Cope

Despite these issues, participants recognized and acknowledged that diabetes is lifelong and that acceptance of this was important for ongoing self-management:

*“So I don’t see it as our fault, but that’s what we have to do.”*

One way that participants learned to cope was by bending the rules to help with maintenance and motivation, recognizing the mental health toll of being too obsessed with adherence:

*“I believe that if you totally rule stuff out, or for me personally anyway anything that you totally rule out becomes a different type of obsession.”*

### 3.4. Theme 4: Self-Management

#### 3.4.1. Competing Demands

Trying to integrate self-management of diabetes into everyday life was reported as a major challenge by participants. This was especially the case when participants lived with other family members:

*“And a lot of times you fall down because family has to live as well. And family cannot just design their life around your diabetes.”*

Fitting diabetes into a busy lifestyle was also acknowledged as challenging:

*“Fitting it all into what isn’t a Monday to Friday 9 to 5, its, diabetes is with us all the time. And many of us have lifestyle patterns that don’t allow for this regulatory of self-management …”*

The cost of self-management was also noted by several participants to be a barrier to self-management:

*“Things like the cost of a good, a good diet…which is a big thing, I think.”*

#### 3.4.2. Taking Responsibility

Participants identified that they needed to take responsibility for working out what the best self-management approach is for them:

*“Well, I’ve tried low carb, high protein, fasting, every different diet that you can think of. And eventually you work out that as long as you do what’s right for you for the most part, then you’ll keep things under control.”*

A key driver for taking responsibility for maintaining healthy behaviors was the fear of consequences if their diabetes became uncontrolled:

*“So the thing is…once a diabetic always a diabetic. We won’t have our arms chopped off, legs chopped off, whatever. But if we let it get out of control, it will control us, that is the problem.”*

Participants admitted to bending the rules here and there and finding a balance that works for them over time. Others have developed habits that help them stay in line:

*“It’s interesting because every one of us is different, myself I do quite a few kilometres and I’m out and about continually. And I found out this rye dry biscuit is ideal for me when I drive, when I want to have something in my stomach and to feel that my stomach is full. I have a couple of those, and I drink coffee that’s another that I’ve got. And that’s the only thing that keeps me from going to the local McDonalds or something or getting food and craving for food or anything.”*

#### 3.4.3. Behavior Change

Participants acknowledged that turning knowledge into sustained behavior change is hard:

*I know that exercise is very good, I’ve learnt that through education, but doing it every day, eating good foods. Again, that maintenance part is the hardest.”*

People reported being consistent for a period of time, but then lapsing:

*Well, knowing the rules is one thing, but the problem is behaviour, this is what I found myself. That when I behave to the knowledge that I’ve got because I might be consistent for a week, 2–3 weeks, but as bugger it, let’s, I’m going, I’m going well and then I forget about it, then I need to redo it again at somewhere else.”*

To counteract this, participants described self-management strategies they used to help them stick with dietary or medication regimes:

*“But just, just simple things like that, even if you just put a note on your fridge saying, no more sweets which is what I think people do in between meals…”*

#### 3.4.4. Apps and Use of Technology

About half of the participants had previously used apps or other digital technologies to help with self-monitoring and adherence. For those who had no experience, barriers were seen as a lack of trust:

*“You can get conflicting apps telling you different things and I, I think there’s that, I don’t know for me it’s more, if I can see it, touch it, feel it, I can feel more confident.”*

Another barrier was that participants did not perceive that there was a need for digital technologies:

*“And for me, I let my body teach me, what is good for me, what is not.”*

For those participants who had used apps, including interactive elements, it was seen as very valuable:

*“And the dietician gives us updates and we can ask questions. So it’s very interactive as well and we have lots of lessons, they’re online as well, on the website. So it’s very informative and very up-to-date as well.”*

## 4. Discussion

This study demonstrates the important role of a CRG in identifying unmet needs in the self-management of T2D by individuals with lived experiences, including those from a CALD background. The findings from the qualitative analysis reflect critical challenges, including ambiguous information or conflicting guidance by health professionals, limited access to tailored guidance, and difficulties in balancing self-management with everyday responsibilities. These issues are consistent with the existing literature, which emphasizes the complexity of managing T2D, where cultural, psychosocial, and systemic factors interplay [11,14,18].

A key finding was the ambiguous or conflicting guidance from health professionals for T2D management. Consumers expressed a preference for support primarily from diabetes educators and nurses, who were perceived as well equipped to address their needs. This aligns with recommendations in the 2019 American Diabetes Association (ADA) and European Association for the Study of Diabetes (EASD) consensus report, which advocates for patient-centered care led by specialists in diabetes management [11]. On the contrary, the DAWN2 study reported that insufficient support from healthcare providers contributes to patients’ reliance on external, often conflicting resources [14]. In this regard, establishing a collaborative multidisciplinary team of medical specialists and allied health professionals, including endocrinologists, GPs, diabetes educators/nurses, dietitians, and psychologists, is crucial for effective diabetes management. Additionally, implementing ongoing training programs for GPs and primary healthcare providers is essential to enhance diabetes-related guidance. This holistic approach has previously been shown to improve glycemic control, reduce the risk of complications, enhance patient satisfaction, and lower healthcare costs [39].

The dietary challenges faced by CALD consumers were also a prominent subtheme, since they reported difficulty accessing culturally appropriate dietary advice. For instance, those with a South Asian or Middle Eastern background expressed frustration with the lack of vegetarian, low-carb diet options, highlighting a critical gap in dietary management resources that fit their dietary needs. This is consistent with existing research, which identifies communication barriers and a lack of culturally tailored health resources as significant obstacles in T2D management for CALD people [22,40]. In the same context, Fitzgerald et al. [23] also reported that as national guides to healthy eating mostly refer to the general population within a country, they fail to cover the needs of CALD and other minority populations, which further emphasizes the need for healthcare systems to provide more culturally specific dietary interventions. 

Insufficient mental health support was another critical unmet need, with participants reporting feelings of frustration, hopelessness, and guilt in their constant fight to manage their condition. This aligns with findings from the DAWN2 and DIABASIS studies, which underscore how emotional distress can negatively impact diabetes self-management [14,18]. In this regard, research has consistently shown that mental health support is vital in facilitating effective diabetes management, particularly in patients with chronic stress. The lack of mental health support in T2D care further challenges people with T2D, highlighting the need for a more holistic approach that also addresses their psychological needs, which on most occasions are left completely unsupported [14,15,16,17]. Therefore, it is crucial to establish collaborative care models with mental health professionals and to train diabetes care providers to recognize and address mental health issues. Ensuring easy access to behavioral health services, offering patient education and support groups, developing personalized care plans, and leveraging digital tools with mental health resources could potentially lead to better adherence to management plans and patient overall well-being [41].

Consumers also expressed their concerns with regard to the food industry’s influence on public perceptions of diet and diabetes. This aligns with broader evidence on the commercial determinants of health, which refer to the strategies used by industries—including food and beverage companies—to shape consumption patterns and health outcomes through marketing, product placement, and lobbying efforts [42]. The food industry plays a significant role in shaping food availability and preferences, particularly through aggressive marketing of unhealthy products, which can contribute to poor dietary habits and exacerbate chronic conditions like T2D. In line with this, participants reported the challenges of navigating misleading food labeling and marketing that often promote unhealthy products as suitable for diabetes management. These commercial influences can undermine public health efforts and lead to confusion among consumers regarding appropriate dietary choices. Misinformation from food marketing and conflicting dietary guidelines were cited as significant barriers to effective self-management, particularly regarding sugar intake and carbohydrate consumption. This mirrors findings from Buckley’s study [22], which highlights the role of industry-driven narratives in shaping public misconceptions about diet and chronic disease management. Therefore, addressing the commercial determinants of health is critical in creating a healthier food environment, especially for vulnerable populations like those living with T2D. To counteract food industry-driven misinformation, future interventions need to focus on stricter regulations on food marketing, clearer food labeling practices, and public health campaigns targeted specifically at T2D patients to increase awareness and help them make informed dietary choices.

In addition to the concerns surrounding the food industry, consumers also emphasized the critical role of family support in managing T2D. Family involvement often plays a central role in dietary choices, emotional support, and ensuring adherence to treatment regimens. Participants mentioned that support from family members was instrumental in helping them make healthier food choices and stay motivated to manage their condition. This finding aligns with previous research, which suggests that strong familial relationships are associated with better disease management outcomes, particularly in chronic conditions like diabetes [43]. 

Similarly, the role of technology in diabetes management was another important sub-theme identified. Many participants highlighted the usefulness of digital tools, such as mobile health apps, wearable devices, and online support communities, which have been shown to enhance self-management and improve glycemic control. For example, technology allows for easier tracking of dietary intake and blood glucose levels, which helps users make informed decisions about their food choices and physical activity. This aligns with previous evidence that has demonstrated the benefits of using technology, including mobile health apps and continuous glucose monitoring systems. These could improve diabetes self-management by facilitating real-time tracking of blood glucose and dietary intake, enabling patients to make more informed decisions regarding their food choices and physical activity [44,45]. However, for those who never used technology to manage their conditions, the main barrier was a lack of trust in technology and a perceived lack of necessity for digital tools in T2D management. This concern is supported by studies indicating that inconsistencies in app accuracy and the absence of standardized regulations for health apps contribute to user distrust [46]. Additionally, digital health disparities, particularly among older adults and socioeconomically disadvantaged groups, have been documented as major barriers to the widespread adoption of these technologies [47]. Access to reliable, user-friendly, and culturally appropriate digital tools, combined with education on their use, is critical to overcoming these challenges and ensuring that the benefits of technology in diabetes management are fully realized.

Most importantly, consumers identified strategies for improving self-management of their T2D. These included enhanced training for primary care providers, a more prominent role for diabetes nurse specialists, and greater commitment from diabetes organizations in delivering culturally relevant education and other resources. These suggestions align with recommendations from existing research, which advocates for more tailored and comprehensive models of care that also provide mental health support, dietary counseling, and patient-centered communication [11,13]. 

One of the strengths of the study is its qualitative design, which allowed a deep exploration of participants’ lived experiences on barriers and facilitators of T2D self-management that could inform the development of targeted interventions. Additionally, the development of the CRG ensured that consumer voices were included, enhancing the validity of the findings. The training workshop conducted prior to the focus groups was another strength, preparing participants and improving the quality of their contributions by ensuring they were familiar with the key themes and questions. However, like most qualitative research, the findings may have limited generalizability due to the small sample size, regional focus (participants were recruited from a single geographic area), and language restrictions (English speaking only). Therefore, the diverse experiences of individuals across different regions, including Indigenous Australian communities or healthcare settings, may not have been fully captured. Additionally, the study sample primarily consists of older adults with T2D, which may not reflect the perspectives of younger individuals. Also, while many participants favored a low-carb approach, our study does not advocate for any single dietary intervention. Instead, we highlight that participants expressed frustrations with conflicting dietary advice from health professionals and a lack of personalized guidance. Finally, the group dynamic in focus group discussions may have influenced the findings, with certain voices potentially dominating the conversation. 

Nonetheless, this study provides crucial insights into the specific needs of individuals with T2D and highlights important areas for future research and intervention co-design. Future studies should aim to expand representation to capture a wider range of experiences and perspectives, to better understand the unmet needs of people living with T2D. Additional recruitment strategies are required to enhance inclusivity, including translation of study materials into multiple languages, recruitment through community health centers and culturally specific diabetes organizations, and engagement with primary care providers to reach underrepresented groups. Quantitative research is also warranted to measure the prevalence of unmet needs across a larger, more representative sample. Follow-up studies are required to evaluate the long-term impact of the CRG’s recommendations on patient care. These may include tracking participant engagement in co-designed intervention programs, monitoring changes in self-management behaviors post-study, and collaborating closely with healthcare providers to assess the adoption of CRG-driven recommendations.

## 5. Conclusions

In conclusion, this study managed to establish a research-ready CRG that was trained to lead the co-design of interventions for the self-management of T2D. It also highlighted the critical unmet needs of people with T2D. Through two focus groups, the study uncovered critical barriers, including the lack of culturally tailored dietary advice, insufficient mental health support, and inconsistent information from healthcare providers. Participants also raised concerns about the influence of the food industry on public perceptions of diet and chronic disease management, as well as challenges with accessing and trusting technology for self-management. These findings highlight the importance of actively engaging individuals with lived experiences in the co-design of interventions to ensure that healthcare systems are responsive to the unique needs of diverse populations. Future research should focus on evaluating consumer-driven models of care that incorporate cultural sensitivity, mental health integration, and more robust patient-provider interaction to enhance T2D management.

## Figures and Tables

**Table 1:** Themes and sub-themes in relation to unmet needs for self-management of T2D.

Theme	Sub-Theme	Example Quote
Need for guidance	Role of health professionals	*“Finding GPs who have time and knowledge to tackle this issue is a major problem.”*
	Role of diabetes Associations	*“We’re carbohydrate intolerant, but they are recommending 5 serves of carbs a day.”*
	Diet management	*“Information about this is non-existent, right. Vegetarian, no carb diet”*
Ambiguous and conflicting information	Information from health professionals and other sources	*“Doctor one time say to me, you only need to check your glucose once a week”*
	Diet misconceptions	*“I don’t think I can live the rest of my life with no potato, rice, bread”*
	The food industry	*“[food] labelling is a nightmare”*
Mental Health	Managing negative emotions	*And so, the hopelessness is that I am going to get amputation, or I am likely to get heart disease, or I am likely to get Alzheimer’s or dementia. And there is nothing I can do.”*
	Role of others	*“So, I often tell people that, I have got diabetes…But they really have no understanding what it is.”*
	Learning to cope	*“So, I don’t see it as our fault, but that’s what we have to do”*
Self-management	Competing demands	*“…family cannot just design their life around your diabetes”*
	Taking responsibility	*“…eventually you work out that as long as you do what’s right for you for the most part, then you’ll keep things under control.”*
	Behavior change	*Well, knowing the rules is one thing, but the problem is behaviour, this is what I found myself.*
	Apps and the use of technology	*“You can get conflicting apps telling you different things”*

## Data Availability

The data presented in this study are available on request from the corresponding author (due to participant confidentiality).

## Supplementary Materials

The following supporting information can be downloaded at: https://www.mdpi.com/article/10.3390/nu17071243/s1, File S1: Involving People in Diabetes Research.

## Abbreviations

The following abbreviations are used in this manuscript:

ADA	American Diabetes Association
CALD	Culturally And Linguistically Diverse
COREQ	Consolidated criteria for reporting qualitative research
CRG	Consumer Reference Group
EASD	European Association for the Study of Diabetes
GP	General Practitioner
IRF	Interim Reference Group
QoL	Quality of Life
T2D	Type 2 Diabetes
TOR	Terms Of Reference
